# When Pain Catastrophizing Is Not Catastrophizing: Identifying Normative From Exaggerated Responses Relative to Referent Pain Intensity

**DOI:** 10.1155/prm/8839902

**Published:** 2025-11-03

**Authors:** Laura A. Frey-Law, Jennifer E. Lee, Adam Janowski

**Affiliations:** ^1^Department of Physical Therapy & Rehabilitation Science, Carver College of Medicine, University of Iowa, Iowa City, Iowa, USA; ^2^Counseling Department, School of Behavioral and Social Sciences, St. Edward's University, Austin, Texas, USA

**Keywords:** mixed linear models, negative affect, negative emotionality, Pain Catastrophizing Scale (PCS), pain intensity, pain rating schema, repeated measures, state catastrophizing, trait catastrophizing

## Abstract

**Background:**

Although pain catastrophizing has been studied widely, there is no consensus on what constitutes an exaggerated response, that is, true catastrophizing, from what might be proportional unpleasant or negative responses to pain. Most available catastrophizing assessments ask respondents to consider when “in pain,” with no assessment of these referent pain anchors. Thus, the influence of referent pain on catastrophic thinking remains unclear. We aimed to assess consistency across referent pain scenarios and to characterize “high” catastrophizing—representing exaggerated responses relative to referent pain intensity.

**Methods:**

A total of 228 adults (152F) completed this observational study. The Pain Catastrophizing Scale (PCS) was completed 4 times interspersed with other assessments. First, with standard instructions, then with specific referent scenarios in a blocked order to minimize order effects. Anticipated scenario pain intensities were rated using a 0–10-cm scale. PCS cross-situational consistency was assessed with intraclass correlations. Mixed linear models evaluated the PCS—referent pain relationship, with and without covariate adjustment.

**Results:**

PCS cross-situational consistency was high, with ICCs = 0.79–0.84. However, total scores varied significantly across referent scenarios, where catastrophizing generally increased with referent pain intensity (*R* = 0.74, *p* < 0.0001), and pain explained 40% of PCS variability. The best fit model of “high” catastrophizing, using the 75th percentile, varied with referent pain intensity, underscoring the importance of contextual anchors, without notable sex differences.

**Conclusions:**

Trait PCS scores should not be interpreted as context-free indices of catastrophizing. The wide range of published PCS cut points may in part reflect differences in referent pain, highlighting the need to contextualize catastrophizing scores for appropriate interpretation.

## 1. Introduction

Catastrophizing has been defined to maladaptive and exaggerated cognitive and emotional responses to actual or anticipated pain [1–3]. Others have further clarified the definition as “to view or present pain or pain-related problems as considerably worse than they actually are” (Convoy, 2020). These somewhat different definitions share an emphasis on a *disproportionate* negative response to, or perception of, pain, which may involve an exaggerated appraisal of threat [3]. Given that pain is “an unpleasant sensory and emotional experience” [4], some degree of negative thoughts and feelings are expected. While debate continues regarding the ideal characterization of pain catastrophizing as a cognitive schema versus a coping strategy [5], or whether it should include constructs of pain-related worry and distress (Ref. Convoy, Crombez), less attention has been given to clarifying what constitutes a *disproportionate* or exaggerated response.

The Pain Catastrophizing Scale (PCS) is widely used; however, there remains no consensus on what score represents a true threshold of catastrophizing, that is, an exaggerated negative response, from what might be proportional given that pain is a negative emotional and sensory experience [4]. Various cut points have been employed in the literature to operationally designate “high catastrophizing” following different strategies. The PCS User Manual suggests 30 is a clinically meaningful cutoff for clinical pain populations [6] and is used by some authors [7, 8]. Others have chosen the 67^th^–75^th^ percentiles from their respective cohorts as “high” catastrophizing, but this has resulted in often widely different cutoff values, for example, 16 to 37 [9–11]. These inconsistencies in single cut-point determinations make between cohort interpretation of pain catastrophizing challenging.

Considered to have both state and trait characteristics [2, 12, 13], situational catastrophizing varies in response to specific pain conditions [14–16], yet has temporal stability with test-retest investigations [17, 18]. Despite observed situational differences, some degree of cross-situational consistency in catastrophizing scores has been reported across different referent pain states, further supporting the trait component of this construct [13, 18, 19]. However, unlike some pain-related constructs, in which multiple scenarios or situations are presented to best ascertain a generalized, trait response (e.g., Fear of Pain Questionnaire or Kinesiophobia), most assessments of catastrophizing do not provide specific situations to enable consistency in contextual interpretation. Instead, catastrophizing assessments typically ask respondents to consider when they are “in pain” without assessing or restricting the choice of referent pain anchor(s). Thus, different referent pain experiences may contribute to interindividual differences in PCS scores, as notable variability in referent pain anchors was observed in those completing the PCS considering pain type (e.g., musculoskeletal, visceral, dental, low back, or headache) [18] or pain recency (i.e., experiences within prior 24 h vs. > 24 h) [20]. Although pain intensity is frequently used as a marker of pain severity or impact, the role of referent pain intensity on measured PCS scores has not been well characterized.

Thus, the aim of this study was to assess catastrophizing response consistency across different referent pain scenarios and to characterize “high” PCS scores, that is, those representing an exaggerated response, as a function of pain intensity, one measurable characteristic of referent pain. We hypothesized that typical endorsement of negative thoughts and feelings using the PCS would show good cross-situational consistency and would generally increase with increased referent pain intensity, enabling “high” scores to be better characterized contextually by referent pain intensity. Secondary aims included examination of catastrophizing subscales, influence of additional covariates, and sex differences. This information may provide a first step to improving discernment of what constitutes truly exaggerated negative pain-related thoughts and feelings versus normative negative responses when considering one characteristic of referent pain anchors.

## 2. Methods

Participants were recruited from the local and university community to complete a series of pain-related surveys at a single visit. Inclusion criteria were intentionally minimal to encourage a wide range of participants, that is, adults 18 years and older. Participants were excluded for inability to read or understand English fluently. All participants provided written informed consent as approved by the University of Iowa Institutional Review Board (#200801772) prior to survey completion and were reimbursed for their time. To be adequately powered to perform regression analyses with one primary predictor and several covariates (e.g., up to 10), we aimed for a sample size of at least 200 participants.

Using a within-subject, experimental study design, individuals first completed the PCS [17] using the standard instructions (i.e., “when you are in pain”), which does not define the recalled, referent pain condition(s) used by respondents. Between other surveys collected and used as covariates, participants completed three additional assessments of the PCS with modified instructions to specifically consider a unique referent pain scenario for each (additional details below) in a blocked, balanced order. The additional questionnaires included (1) participant demographics; (2) the Positive and Negative Affect Schedule (PANAS) [21]; (3) the Pain Schema Inventory (PSI) [22]; and (4) the Somatosensory Amplification Scale (SSAS) [23]. All assessments were completed on paper, with packets pre-assembled to achieve the blocked, balanced order of six permutations in roughly groups of 10 (i.e., 1-2-3; 1-3-2; 3-2-1; 3-1-2; 2-1-3; and 2-3-1) throughout recruitment to achieve approximately equal proportions. However, the packet permutations were not recorded as a variable, so we do not know the exact distribution of the final cohort, as packets were disassembled as part of the data scoring and entry process.

### 2.1. Participant Characteristics

Participants reported sex (M/F), age, race, ethnicity, general health status, current pain, and “most intense physical pain” ever experienced. Pain intensity was assessed throughout the study using a horizontal, 10 cm visual analog scale (VAS) with anchors from “no pain” on the left to “maximum pain” on the right [24].

### 2.2. PCS

The 13-item, standard PCS was used to assess catastrophizing [17]. It instructs participants to “indicate the degree to which you have these thoughts and feelings when you are experiencing pain.” No instructions for which referent pain experience(s) to consider are provided. Each item is scored using a 5-point scale, ranging from 0 (*not at all*) to 4 (*all of the time*). The three PCS subscales are rumination (range 0–16), magnification (range 0–12), and helplessness (range 0–24), which when summed produce a total score (range 0–52). The PCS has shown high internal consistency (0.87) and high test-retest reliability (0.75) [17].

For the modified PCS instructions, participants were asked to consider each of three referent pain scenarios, first rating the anticipated pain intensity and then completing the PCS. The three referent pain scenarios were loosely adapted from similar items on the Fear of Pain Questionnaire [25], targeting mild, moderate, and severe acute pain that was easily imagined or previously experienced based on pilot testing in a separate cohort. As catastrophic thinking may be expected to be greater in response to chronic pain, acute pain scenarios were chosen to provide a conservative estimate of catastrophizing as it relates to pain intensity, as follows:

1. Foot splinter: “Imagine getting a small splinter in your foot that you cannot remove”; (targeting mild pain)
2. Shin strike: “Imagine while hurrying through your home, you strike your shin on the corner of a coffee table.” (targeting moderate pain), and
3. Hand in door: “Imagine someone slams a car door on your hand.” (targeting severe pain).

In addition to total PCS scores for each scenario, subscale scores were evaluated. As each subscale has a different range, standardized scores, that is, mean item scores with a range of 0–4, were also computed to make comparisons between subscales and with total scores more easily interpretable.

### 2.3. Covariates

In addition to the demographic characteristics described above, several surveys were collected for use as additional covariates. Negative affect (NA) and positive affect (PA) were assessed using the PANAS [26, 27], a validated self-report trait measure [21]. Multisensory sensitivity was assessed using the SSAS [23, 28]. The PSI was assessed, in which respondents rate expected pain intensities for six painful conditions at three severity levels: “mild,” “moderate,” and “severe” [22, 29]. Mean pain ratings from the 18 PSI items were used to adjust for individual differences in pain rating schema [30, 31].

These covariates—sex, age, presence of current pain, maximum pain experienced, mean pain schema, SSAS, PA, and NA—were selected based on theoretical relevance and prior empirical findings suggesting their influence on pain perception and reporting. Sex and age are commonly associated with differences in clinical pain prevalence and pain sensitivity and thus were included to account for demographic variability. The presence of current pain and maximum pain experienced were included to control for individual differences in pain history and current pain status, which could influence responses to hypothetical pain scenarios. The mean pain schema reflects cognitive representations of pain and was included to account for individual differences in pain conceptualization, particularly relevant to the pain intensity ratings of each scenario. SSAS was included as a measure of multisensory sensitivity, which may modulate perceived intensity of pain. PA and NA were included to account for affective states that can influence pain perception and reporting. Particularly NA has been related to PCS, as they both reflect negative valence traits.

### 2.4. Statistical Analyses

Summary statistics for all variables, including means (standard deviations) and frequencies (percent), were calculated using SPSS v28.0.1.1 (IBM). Differences in referent pain intensities across the three scenarios (foot splinter, shin strike, hand in door) were assessed using repeated measures analysis of variance (RM ANOVA), unadjusted and adjusted for sex (M = 0, F = 1), age, presence of pain (Y = 1, N = 0), maximum pain experienced, mean pain schema, SSAS, PA, and NA. Standard (no referent pain defined) and scenario-specific PCS scores were also compared using RM ANOVA, both unadjusted and adjusted considering the above covariates. Covariates were included to ensure that observed differences in referent pain intensities and PCS scores across scenarios were not confounded by these individual differences. This approach aligns with our hypothesis that scenario-specific pain ratings and catastrophizing scores may vary independently of these covariates. By evaluating both unadjusted and adjusted models, we are better able to interpret any observed scenario differences as independent or in part explained by the assessed covariates. For significant differences between scenarios, effect sizes were computed (Cohen's *d*).

#### 2.4.1. Cross-Situational Consistency

To assess cross-situational consistency, two-way random effects intraclass correlation coefficients (ICCs) for average measures were evaluated for the three scenario-specific PCS scores, with and without the standard PCS. Interpretation of ICCs was operationally defined as fair (0.3 to < 0.5), moderate (0.50 to < 0.75), good (0.75 to < 0.9), and excellent (0.9 and greater) cross-situational consistency [32].

#### 2.4.2. Unadjusted Catastrophizing—Pain Intensity Relationship

To evaluate the relationship between catastrophizing and referent pain intensity, mixed linear models (i.e., repeated measures regression) were employed using the *lmer* function from the lme4 library in the R Statistical Package (v4.2.2) using RStudio (v2022.12.0+353). The repeated outcome variable was the scenario-specific PCS score, with their corresponding referent pain intensities as fixed predictor variables (i.e., unadjusted model). Mixed models allow for fixed and random (i.e., participant-specific) factors to be included. Both random intercepts and slopes were considered. Estimated *R*^2^ values were determined using the *r.squaredGLMM* function in *R*. This analysis provides two *R*^2^ values: (1) marginal *R*^2^ (m*R*^2^) and (2) conditional *R*^2^ (c*R*^2^). The m*R*^2^ represents the outcome variance (e.g., PCS score) explained by the fixed effects in the model (i.e., pain intensity for the unadjusted model); whereas the c*R*^2^ represents the total outcome variance explained by both the fixed and random (i.e., participant-specific) factors [33]. A larger discrepancy between the two *R*^2^ values indicates greater inter-individual variation in the relation being modeled. Conversely, when the two *R*^2^ values are relatively similar, the relationship between the outcome and its predictor(s) is relatively similar across individuals. Repeated measures correlations (rmcorr) between scenario-specific PCS scores and referent pain intensity were also evaluated and used to create scatterplots of the data [34].

#### 2.4.3. Modeling “High” Pain Catastrophizing

The unadjusted mixed linear model, representing PCS relative to referent pain intensity, was used to determine normative percentiles. That is, “high” PCS values were operationally defined as greater than or equal to the 75^th^ percentile using the participant-specific random factor(s).

#### 2.4.4. Adjusted Catastrophizing—Pain Intensity Relationship

To explore which covariates were significantly related to the PCS-pain intensity relationship, two adjusted models were considered. First, one in which all possible covariates were added to the unadjusted mixed linear model predicting catastrophizing responses as a function of referent pain intensity. These included the following categorical and continuous fixed variables: age; sex and sex interactions; PSI; NA; PA; SSAS; maximum prior pain; and presence of current pain. A second adjusted model was evaluated in which the standard PCS score was also included.

#### 2.4.5. Exploratory PCS Subscale Analyses

The above mixed linear models were repeated for each of the PCS subscales as the outcome variable: rumination, magnification, and helplessness. Both unadjusted and adjusted models were evaluated.

## 3. Results

### 3.1. Study Participants

A total of 229 adults were enrolled in the study and included in the assessment of cross-situational consistency. However, one participant did not complete the demographics survey; thus, all demographic characteristics are missing. An additional 37 participants' referent pain intensity ratings for two or more of the three scenarios were missing and thus were excluded from the analyses involving pain ratings. These missing data were due to the omission of the pain VAS line in some early survey packets and others who simply missed marking the line. The cohort ranged in age from 18 to 55 years (mean 27.8); was 67% female (*n* = 151/226), 33% male (75/226) (2 did not indicate their sex), and was predominantly Caucasian (88%, 199/226; 2 did not report their race or ethnicity) (see Table 1). Approximately 32% indicated having some pain at the time of the study (72/227), and nearly all reported having good (120/222, 54.1%) or excellent (96/222, 43.2%) health.

### 3.2. Scenario Comparisons

**Figure 1:**
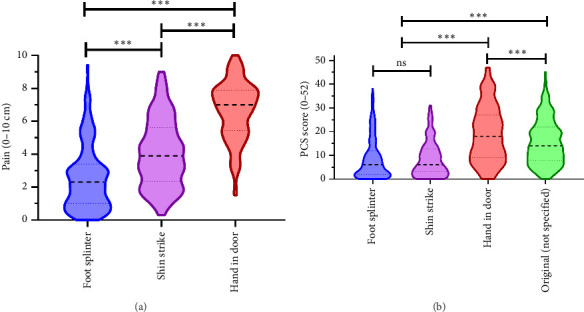
Violin plots of referent pain intensity and corresponding PCS scores. The three referent pain scenarios, removing a splinter from your foot, striking your shin on a table, and slamming your hand in the door, resulted in (a) low, moderate, and high pain intensity ratings that differed significantly from each other (*p* < 0.001) and (b) PCS scores that were each different from the standard PCS score (with no directed referent pain) and from each other, except the foot splinter and shin strike scenarios. ^∗∗∗^*p* < 0.001, ^∗∗^*p* < 0.01, and ^∗^*p* < 0.05.

The referent pain ratings differed significantly by scenario with (*F*_2,364_ = 11.9, *p* < 0.0001) or without adjustment for covariates (*F*_2,384_ = 442.4, *p* < 0.0001) with no significant scenario-covariate interactions (*p* = 0.10–0.89). However, two covariates were related to the overall referent scenario pain ratings (i.e., on average across scenarios): pain schema (*F*_1,182_ = 19.7, *p* < 0.0001) and NA (*F*_1,182_ = 12.8, *p* < 0.0001). Scenario pain ratings were not related to sex (*p* = 0.28), age (*p* = 30), maximum pain experienced (*p* = 0.56), or whether they were currently experiencing pain (*p* = 0.67). Follow-up post hoc tests indicated the foot splinter scenario had the lowest unadjusted pain rating (mean 2.5, SE 0.14), followed by the shin strike (4.0, SE 0.15), with the most painful scenario being slamming a hand in the door (6.7, SE 0.13), all with *p* values < 0.001 with Bonferroni correction (see Figure 1(a)). Cohen's *d* effect sizes ranged from 0.7 to 2.0, confirming the three scenarios were perceived as different from one another regarding pain intensity.

PCS scores differed between the original instructions and the three scenarios for the unadjusted (*F*_3,681_ = 166.0, *p* < 0.0001) analyses. Follow-up post hoc tests revealed that all pairwise comparisons were significant (*p* < 0.001) except between PCS scores for the foot splinter and shin strike scenarios (8.4, SE 0.49 vs. 8.5, SE 0.57, *p* = 1.0). The original PCS score was higher than these first two scenarios (15.5, SE 0.62) but less than the hand in door scenario (18.9, SE 0.77) on average (Figure 1(b)). Effect sizes for the significant pairwise comparisons ranged from *d* = 0.35 to 0.93.

### 3.3. Cross-Situational Consistency

The ICCs for the PCS scores across the three scenarios were 0.84 and 0.79, with and without the original PCS, respectively. Thus, pain catastrophizing showed good cross-situational consistency across the defined and undefined referent pain conditions.

### 3.4. Unadjusted Catastrophizing—Pain Intensity Relationship

The linear mixed models of PCS—pain intensity relationships did not converge with random slopes; thus, only random intercepts were included. The unadjusted PCS—pain model showed a significant relationship between referent pain intensity and PCS score (see Table 2). The fixed factor relationship is represented as a function of pain intensity (equation (1)), where for each 1 cm increase in referent pain intensity (i.e., a 10% increase on a 10 cm VAS), PCS scores increased on average 2.6 points (out of 52, a 5% increase).

(1) $$\begin{array}{c} \text{PCS}=0.2+2.63*\left(\text{Pain Intensity}\right). \end{array}$$

Further, the PCS model fixed intercept was not significantly different from zero (intercept = 0.20, SE = 0.70, *p* = 0.77), indicating that, on average, PCS scores approach zero as referent pain intensity approaches zero. This unadjusted linear model predicts that referent pain intensities of 3, 5, and 9 out of 10, for example, correspond to average PCS scores of approximately 8, 13, and 24 on average.

**Figure 2:**
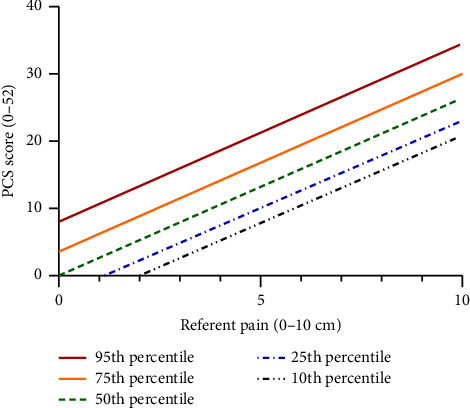
Modeled linear relationship between referent pain intensity and PCS score for 10th through 95th percentiles. Normative PCS scores are represented as a function of referent pain intensity, based on mixed linear models with random intercepts to account for individual differences. The dashed blue and black lines represent catastrophizing scores based on percentiles for low (10th and 25th); the green dashed line is average (50th), and the solid orange and red lines depict high (75th and 95th) catastrophizing as a function of referent pain intensity. Note that the same total PCS score may be considered “exaggerated” for a low referent pain intensity yet typical or even below average for a high referent pain intensity.

The marginal and conditional *R*^2^ values for the unadjusted total PCS—pain intensity model were 0.40 and 0.68, respectively (Table 2), indicating 40% of the variance in PCS scores was explained by referent pain intensity. Further, the 0.28 difference between m*R*^2^ and c*R*^2^ indicates this relationship showed moderate between-individual heterogeneity. The repeated measures correlation across scenario-specific total PCS scores versus pain intensity was also high at *R* = 0.74 (95^th^ confidence interval 0.69 to 0.78, *p* < 0.0001). See Supporting Information Figure S1 for a scatterplot showing the fitted correlation lines and data points for each participant. The random model intercepts for the unadjusted linear model, representing PCS scores by percentiles, are provided in Supporting Information Table S1. The resulting linear model representations of PCS scores relative to referent pain intensity only for the 10^th^, 25^th^, 50^th^, 75^th^, and 90^th^ percentiles are shown in Figure 2 and relative to reported PCS cutoff values from the literature in Supporting Information Figure S2.

### 3.5. Modeling “High” Catastrophizing

We use the term “high catastrophizing” to describe high scores on the PCS in the context of referent pain intensity, while recognizing that this does not always mean a response is exaggerated—since we do not yet have a clear way to define what counts as exaggeration. Instead, we chose to use the 75^th^ percentile from the mixed linear model as an operational definition for an “exaggerated” PCS response (Table S1). That is, we shifted the above model representing the average PCS-pain intensity response (equation (1)) by adding the random intercept value for the 75^th^ percentile (i.e., 3.65) rather than the mean intercept representing the 50th percentile. As such, equation (2) represents “high” PCS as it relates to referent pain intensity—providing a contextual anchor rather than a single cut point to interpret PCS scores. Again, considering example referent pain intensities of 3, 5, and 9 out of 10, this model estimates corresponding “high PCS” score thresholds to vary from roughly 12, 17, and 28, respectively.

(2) $$\begin{array}{c} \text{High total PCS score}=3.85+2.63*\left(\text{Pain Intensity}\right). \end{array}$$

### 3.6. Covariates Influencing the Catastrophizing–Pain Relationship

When exploring which covariates beyond pain helped explain the observed variability in the PCS-pain intensity relationship, a few reached significance for total PCS (Table 3) or subscale models (Table S2). Higher prior maximum pain was related to less catastrophizing (−0.84, *p* = 0.005), with or without adjustment for standard PCS scores (i.e., without any defined referent pain context). Whereas, excluding the standard PCS in the model, higher multisensory sensitivity and NA were both related to higher catastrophizing (*p* = 0.0008 and 0.01, respectively). Sex did not meet our a priori alpha value of 0.01 (*p* = 0.04) in either adjusted model but was closer to reaching significance when including the standard PCS as a covariate. However, the covariates only minimally altered the PCS—referent pain intensity relationship, with beta values of 2.45 and 2.57 on average in the adjusted models and 2.63 in the unadjusted model. Details are provided in Table 3.

### 3.7. PCS Subscale Analyses

When repeating the unadjusted mixed linear models for each catastrophizing subscale, referent pain intensity continued to be a significant predictor, but to different degrees for each (see Table 2, Supporting Information Figure S3). For example, a 1 cm (10%) increase in pain intensity produced the largest increase in ruminating (0.26 per item; 6.5% increase), followed by helplessness (0.19 per item; 4.8% increase), and least magnifying (0.15 per item; 3.8% increase).

When evaluating the covariate influences on these linear models, particularly those without the inclusion of baseline PCS subscales, several differences were noted (see Supporting Table S2). First, ruminating was most influenced by prior maximum pain, where higher prior pain was associated with less ruminating (*p* = 0.003). Second, higher NA was associated with greater magnifying (*p* < 0.001) and to a lesser degree, greater helplessness (*p* = 0.02), but not ruminating. However, all three subscales were associated with multisensory sensitivity (*p* = 0.02 to < 0.001).

## 4. Discussion

Although hundreds of studies have examined pain catastrophizing, relatively little attention has been given to the choice of referent pain used to anchor these assessments. This study focused on the relationship between PCS scores and one characteristic of referent pain—intensity—to explore normative versus exaggerated negative thoughts and feelings (i.e., “high” catastrophizing) across referent pain conditions. Using a repeated measures design, we confirmed good cross-situational consistency in PCS scores, with individuals differing in their tendency to endorse catastrophic thinking statements relative to normative values across referent anchors. Given that PCS scores generally increased with higher referent pain intensity, explaining approximately 40% of the observed PCS variability, interpretation of catastrophizing must consider the referent pain context. This suggests that applying fixed cutoffs without accounting for referent pain characteristics may lead to misleading conclusions.

As noted by Quartana et al. [2], pain catastrophizing is activated during actual or anticipated painful situations, so while trait catastrophizing may be an important consideration when evaluating pain and its influences from a biopsychosocial perspective [35], its assessment is inherently manifested by real or imagined painful situations. Although evidence suggests state catastrophizing assessments have stronger links to concurrent or subsequent pain outcomes than trait catastrophizing in experimental and clinical studies [15, 36], this may in part be due to inconsistent choice of referent pain. Anecdotally, participants from our prior studies have asked which pain they should consider when completing the PCS, for example, “childbirth or when I hurt my knee?” These inquiries, in fact, helped spark the current study's examination of the relationship between catastrophizing and underlying referent pain conditions.

Limited prior research has explored characteristics of the referent pain considered with trait pain catastrophizing assessment [12, 18, 37]. One study involving patients with chronic pain found roughly half considered the prior 24 h when completing the PCS [12], indicating a 50:50 chance “trait” assessments of catastrophizing reflected their current state. In a college-aged sample, nearly 90% considered only 1 (59%) or 2 (30%) referent pain conditions and found that chronicity was a significant referent pain characteristic for PCS scores [20]. The most similar study to our current investigation also asked individuals to respond to the PCS but with modified instructions to specifically consider back, dental, or headache forms of pain [18]. While in older adults, the type of referent pain did not substantially alter PCS responses, in young adults, dental referent pain produced substantially higher PCS scores. This prior study is in part consistent with our current findings, suggesting good cross-situational consistency is apparent with both predefined and individualized referent pain scenarios when considered relative to other individuals and supports that absolute PCS scores can vary substantially based on choice of referent pain anchor. The influence of referent pain intensity on PCS scores observed here is also in line with catastrophizing decreases observed with pain reduction in response to treatment in patients with rheumatoid arthritis and knee osteoarthritis [13, 38]. Ultimately, this raises the question of what proportion of catastrophizing assessment using current tools, without some consideration of the referent pain characteristics, truly reflects the underlying trait aspects of the construct.

The literature is scattered with reports of different criteria to identify what constitutes “high” catastrophizing based on the study cohort or prior study values. For example, PCS cut points ranging from 16 to 37 have been reported for chronic pain populations (out of a possible score from 0–52) [6–9, 17, 39]. While there has been relatively little discussion in the literature regarding these widely varying cut points, the current results, coupled with the limited work in this area, suggest score inconsistencies may, in part, be due to differences in referent pain anchors. Certainly in cross-sectional studies, catastrophizing is reported to explain from 7% to 31% of the variability in pain intensity [3], yet in the current repeated design, the reverse was observed, where referent pain intensity explained 40% of PCS variability.

Collectively, these findings suggest there is room for improvement when assessing trait-like levels of catastrophizing. While referent pain scenarios are likely to impact catastrophic thinking based on several possible characteristics, including underlying threat [12, 16, 18], our results highlight the importance of pain intensity. The lack of consideration of referent pain in trait catastrophizing assessment unintentionally creates two sources of variability in its appraisal: the choice of referent pain and the underlying “true” tendency to experience catastrophic thinking in response to pain. While the parameters for the current models are specific to the scales used (i.e., PCS and 0–10 pain intensity), the underlying conceptual model is the important consideration, namely that “high” catastrophizing should be considered in the context of the underlying referent pain characteristics. This finding does not imply that pain intensity is the only, or even the most important, factor influencing how individuals evaluate their cognitive and emotional responses to pain; rather, it highlights that pain intensity should be considered among other potentially important contextual influences that may impact perceived threat.

While the current study focused on referent pain intensity, clearly other dimensions of the pain experience likely contribute to the tendency to have exaggerated negative thoughts and feelings about pain. This was evident in the current study where two scenarios—splinter removal and hitting their shin—resulted in no difference in PCS scores despite significantly different anticipated pain intensities. The idea of trying to remove a difficult splinter, potentially with a needle, activated relatively greater catastrophic thoughts than did simply hitting their shin on a coffee table. In young adults, a similar finding was reported in which PCS scores were notably higher for dental referent pain compared to back or headache pain, despite similar pain intensity ratings [18]. These results indicate that additional pain qualities beyond intensity influence the tendency to catastrophize, but not all may be as easily quantified. Certainly, a notable portion (i.e., 40%) of the variance in PCS scores was explained by pain intensity alone, increasing to 68% when including individual differences in intercept value. Yet that leaves over 30% unexplained PCS variance that may be influenced by other referent pain characteristics such as chronicity, explained versus unexplained pain, type of pain, prior resolution of similar conditions, among others. We recognize the current model identifying high catastrophizing as a function of pain intensity may be a noteworthy advancement over single cut-point values yet remains a unidimensional model that likely would benefit from future efforts to consider additional referent pain characteristics.

Despite the understanding that not all negative pain-related thoughts and feelings reflect an exaggerated or catastrophic reaction, we currently lack clear vocabulary to describe the full scale of this response. That is, there is no terminology to describe the opposite of catastrophizing (a form of stoicism, perhaps?); nor is there an appropriate description for endorsing “average” negative thoughts and feelings in response to pain. Our findings support that not all estimates of pain catastrophizing, that is, scores above some operationally defined cut point, are truly an exaggerated negative response to pain. Endorsement of negative statements, such as “I wonder when it will end”; or “I cannot take it anymore”; may be appropriate and expected, rather than pathological, for some referent pain anchors. In fact, lack of any negative thoughts or feelings with pain may also be pathologic, but with less clear implications. This lack of a construct vocabulary that adequately communicates the range of possible responses from low to high may be one additional factor that has contributed to the call for revised terminology [40].

Indeed, the terminology of “pain catastrophizing” itself has been questioned, as it may unnecessarily stigmatize patients as having an abnormal or exaggerated response without sufficient contextualization (Ref. Crombex, Convoy, and others). One study determined that many items used to assess catastrophizing across multiple scales may represent pain-related worry or distress more strongly than catastrophizing, as judged by participants (Convoy). Given that no current tools are able to actually characterize any degree of exaggeration (i.e., contextual interpretation relative to norms or self-reported exaggerations), one proposal has been to rename current assessments as measures of pain-related worry or distress rather than catastrophizing (Crombez and Convoy). A change in terminology may be less stigmatizing and have the additional advantage of being potentially less dependent on a specific contextual anchor for directing intervention. Certainly our findings suggest that regardless of the construct label, these assessments using items from the current PCS are influenced by referent pain anchors, but even high “normal” negative responses to pain (i.e., not exaggerated in response to high-intensity pain) may be viable targets for intervention regardless of the referent anchor. An analogy may be treating inflammation following an acute ankle sprain (often a normal response to injury) with compression, ice, and elevation, without labeling the response as exaggerated or abnormal. Consistent with this debate, our findings suggest that the use of single PCS cut-point scores is inherently problematic. Indeed, others have similarly concluded that optimal evaluation of pain catastrophizing, that is, the degree to which it represents an exaggerated response, inherently requires some degree of contextualization and have suggested alternate methods are needed to truly evaluate for an exaggerated cognitive and emotional response to pain (Ref. Crombez 2024 and Convoy 2020).

When considering the influence of covariates on pain and catastrophizing, possibly the most unexpected was the influence of individuals' prior maximum pain intensity ratings on the scenario-specific PCS scores. Those with higher reports of prior maximum pain generally reported lower catastrophizing scores for a given referent pain intensity, yet prior maximum pain had no influence on the scenario pain intensity ratings themselves. This may suggest that negative perceptions of pain may be more modifiable than the cognitive appraisal of pain intensity, based on life experience. Conversely, the pain schema measure, an indicator of how an individual operationally uses the 0 to 10 scale to rate pain [22], was the strongest covariate predicting referent pain intensity, yet had virtually no relationship with PCS scores. Thus, again, showing that while pain intensity and catastrophizing are related, they also demonstrate distinctions that reinforce their uniqueness.

Several limitations are noteworthy when interpreting these findings. First, while the scenarios were chosen based on pilot data for their ability to be relatable by a majority, it is possible not all respondents could rely on past experiences to consider each referent pain scenario. Second, the model between catastrophizing and pain intensity may not universally apply to all forms of referent pain, such as chronic pain or pain of unknown origin. However, we restricted our study to acute pain referent anchors in order to reduce potential confounding by other characteristics. This strategy enabled us to target the relation between catastrophizing and pain intensity across a wide range of individuals, with and without pain, to maximize generalizability. Our sample, while varied by age, sex, and current pain status, was drawn from a largely non-Hispanic White, Midwestern university community, which may limit the generalizability of our findings. Lastly, normative catastrophizing values have been shown to vary somewhat across continents [41]; thus, the model observed here may not fully represent the pain-PCS relationship globally.

In summary, pain catastrophizing is recognized to be an important construct associated with current and future pain outcomes. However, the identification of when negative thoughts and feelings about pain reach a level that reflects true catastrophizing is inconsistent throughout the literature. The results of this study suggest the differentiation of exaggerated from typical or even below-average pain-related negative thoughts and feelings should consider contextual referent pain characteristics, such as pain intensity. This may provide a more nuanced approach to identifying true pain catastrophizing, as not all negative thoughts and feelings in response to pain are indicators of an exaggerated mindset, and thus, not all estimates of pain catastrophizing are actually catastrophizing.

## Figures and Tables

**Table 1:** Mean (SD) (range) or *N* (%) of participant demographic and pain-related characteristics.

	All (*N* = 228)	Females (*n* = 151)	Males (*n* = 75)	Sex difference (*p* value)
Age (years)	27.8 (10.8) [18–55]	27.6 (10.9) [18–55]	26.0 (8.7) [18–54]	0.08
Race/ethnicity (non-Hispanic Caucasian)	199/226 (88.0%)	131/150 (86.8%)	67/75 (89.3%)	0.72
Health (excellent or good)	215/222 (96.8%)	141/147 (95.9%)	74 (98.7%)	0.08
Current pain (yes)	72/227 (31.7%)	48/151 (31.8%)	23/75 (30.7%)	0.86
If yes, intensity (VAS)	3.2 (1.8) [0.1–6.6]	3.4 (1.8) [0.2–6.6]	2.7 (1.8) [0.1–6.2]	0.15
Max prior pain (VAS)	7.5 (1.5) [2.6–10.0]	7.7 (1.5) [2.6–10.0]	7.3 (1.6) [3.1–10.0]	0.15
Standard PCS				
Total (0–52)	15.5 (9.3) [0–45]	16.3 (9.6) [0–45]	14.1 (8.8) [0–35]	0.12
Rumination (0–16)	6.4 (4.0) [0–16]	6.8 (4.0) [0–16]	5.7 (3.8) [0–14]	0.07
Magnification (0–12)	3.6 (2.3) [0–10]	3.5 (2.4) [0–10]	3.7 (2.1) [0–9]	0.65
Helplessness (0–24)	5.5 (4.2) [0–23]	6.0 (4.3) [0–23]	4.7 (4.1) [0–18]	**0.04 ** ^∗^
Negative affect (10–50)	17.7 (5.8) [10–43]	17.4 (5.2) [10–33]	18.0 (6.7) [10–43]	0.45
Positive affect (10–50)	33.2 (6.8) [10–49]	33.4 (6.8) [10–49]	33.0 (6.7) [12–48]	0.64
SSAS (10–50)	25.2 (6.0) [0–43]	25.9 (5.9) [10–43]	24.4 (5.4) [10–36]	0.07
PSI means (0–10)	4.5 (1.0) [1.9–7.0]	4.6 (1.0) [1.9–7.0]	4.4 (1.0) [2.0–6.3]	0.11

*Note:* Two participants did not provide sex information and thus are not included in sex-specific summary data. *p* value for independent *t*-test or chi-square test as appropriate for male/female comparisons, where bold ^∗^denotes *p* < 0.05.

Abbreviations: PCS = Pain Catastrophizing Scale, standard trait instructions; PSI: Pain Schema Inventory, mean of mild, moderate, and severe pain ratings.

**Table 2:** Unadjusted mixed linear model results (*β*, SE, *p* value) for predicting Pain Catastrophizing Scale (PCS) scores (total and standardized mean item) based on referent pain intensity.

Outcome	PCS total scores			PCS—mean item scores			Model *R*^2^
	Score range	Intercept *β*_0_ (SE)	Pain intensity slope *β*_1_ (SE)	Score range	Intercept *β*_0_ (SE)	Pain intensity slope, *β*_1_ (SE)	
PCS total	0–52	0.20 (0.70) *p* = 0.77	**2.63 (0.11)** *p* < 2*e* − 16	0–4	0.02 (0.05) *p* = 0.77	**0.20 (0.009)** *p* < 2*e* − 16	m*R*^2^ = 0.40 c*R*^2^ = 0.68

Ruminating subscale	0–16	**0.80 (0.29)** *p* = 0.006	**1.05 (0.05)** *p* < 2*e* − 16	0–4	**0.20 (0.07)** *p* = 0.006	**0.26 (0.01)** *p* < 2*e* − 16	m*R*^2^ = 0.38 c*R*^2^ = 0.67

Magnifying subscale	0–12	0.42 (0.20) *p* = 0.04	**0.45 (0.03)** *p* < 2*e* − 16	0–4	0.14 (0.07) *p* = 0.04	**0.15 (0.01)** *p* < 2*e* − 16	m*R*^2^ = 0.19 c*R*^2^ = 0.60

Helplessness subscale	0–24	**−0.99 (0.33)** *p* = 0.003	**1.13 (0.06)** *p* < 2*e* − 16	0–4	**−0.17 (0.06)** *p* = 0.003	**0.19 (0.01)** *p* < 2*e* − 16	m*R*^2^ = 0.36 c*R*^2^ = 0.60

*Note:* m*R*^2^ = marginalized coefficient of determination, a measure of variation explained by the fixed factor, pain intensity, only. c*R*^2^ = conditional coefficient of determination, a measure of the total variance explained by pain intensity and a random factor representing individual differences, significant (*p* < 0.01) model beta (SE) values are shown in bold.

Abbreviation: PCS = Pain Catastrophizing Scale.

**Table 3:** Adjusted mixed linear model results (*β*, SE) including covariates with *p* values < 0.05 explaining the variance in the study outcome, total Pain Catastrophizing Scale (PCS) scores, with and without standard total PCS scores (i.e., no predefined referent pain) as a covariate.

Outcome (range)	Adjusted model with standard PCS			Adjusted model without standard PCS		
	Covariate predictor	*β* (SE)	*p* value	Covariate predictor	*β* (SE)	*p* value
PCS total (0–52)	**Referent pain**	**2.45 (0.11)**	**< 2*e* − 16**	**Referent pain**	**2.57 (0.12)**	**< 2*e* − 16**
	**PCS total**	**0.44 (0.04)**	**< 2*e* − 16**	**SSAS**	**0.32 (0.09)**	**0.0008**
	**Max prior pain**	**−0.68 (0.24)**	**0.005**	**Max prior pain**	**−0.80 (0.31)**	**0.005**
	Sex (*m* = 0, *f* = 1)	−1.54 (0.76)	0.04	**NA**	**0.25 (0.10)**	**0.01**
	m*R*^2^ = 0.60, c*R*^2^ = 0.69			m*R*^2^ = 0.47, c*R*^2^ = 0.69		

*Note:* Additional covariates included in the model but with *p* values > 0.05: age, negative affect, mean pain rating schema value, positive affect, Somatosensory Amplification Scale, and presence of current pain (yes/no). All covariates achieving *p* < 0.05 are shown, but only those achieving a priori level of *p* = 0.01 are in bold.

## Data Availability

All study data will be made available upon request from the corresponding author.

## Supporting Information

Supporting Information 1 Supporting Information File 1 includes the following figures and tables: Figure S1: Individual PCS and pain rating data points along with their modeled relationships using random intercepts; Figure S2: Current modeled results relative to previously reported PCS cutoffs used in the literature; Figure S3: Individual PCS subscale mean item scores and pain rating data points along with their modeled relationships using random intercepts; Table S1: Random intercept values for the mixed effects models representing nine levels from the 10th to the 95th percentile; Table S2: Results from the mixed effects models for the PCS subscales.

Supporting Information 2 Supporting Information File 2 contains the STROBE Checklist for this observational study.
