# Supplementary material for: When Pain Catastrophizing Is Not Catastrophizing: Identifying Normative From Exaggerated Responses Relative to Referent Pain Intensity
Source: Pain Res Manag. 2025 Nov 3;2025:8839902. doi: 10.1155/prm/8839902 (PMC12602029; doi:10.1155/prm/8839902)
Supplement: Supporting Information 1 — Supporting Information File 1 includes the following figures and tables: Figure S1: Individual PCS and pain rating data points along with their modeled relationships using random intercepts; Figure S2: Current modeled results relative to previously reported PCS cutoffs used in the literature; Figure S3: Individual PCS subscale mean item scores and pain rating data points along with their modeled relationships using random intercepts; Table S1: Random intercept values for the mixed effects models representing nine levels from the 10th to the 95th percentile; Table S2: Results from the mixed effects models for the PCS subscales. [file 8839902.f1.pdf]

When is Pain Catastrophizing not Catastrophizing?

Supplemental Materials

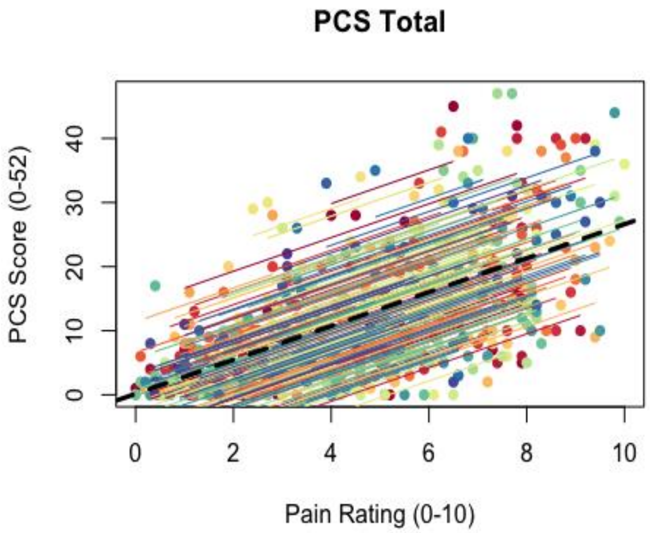

Figure S1. Scatterplot of individual pain catastrophizing scale (PCS) scores relative to their referent pain scenario pain intensities, with the repeated measures modeled correlations for each individual (thin colored limes). The dark, dashed line represents the overall unadjusted relationship between PCS and referent pain intensity, with R = 0.74 (95th percent confidence interval: 0.69 to 0.78, p < 0.00001), using the repeated measures correlation (rmcorr) function in R.

Table S1. Mixed linear model random intercept values (b_i) for the PCS – pain intensity unadjusted models by percentiles, for total PCS and its three subscales: Ruminating (R), Magnifying (M), and Helplessness (H) using both original scale scores and the corresponding standardized mean item scores.

	**Total Scores**				**Standardized Scores**			
**Percentile**	**Total (0 – 52)**	**R (0 – 16)**	**M (0 – 12)**	**H (0 – 24)**	**Total (0 – 4)**	**R (0 – 4)**	**M (0 – 4)**	**H (0 – 4)**
**10th**	-5.41	-1.66	-1.23	-3.17	-0.42	-0.42	-0.41	-0.53
**25th**	-3.46	-0.71	-0.64	-2.25	-0.27	-0.18	-0.21	-0.37
**50th**	-0.12	0.67	0.17	-1.27	-0.01	0.17	0.06	-0.21
**60th**	1.00	1.27	0.51	-0.88	0.08	0.32	0.17	-0.15
**70th**	2.38	1.92	0.90	-0.30	0.18	0.48	0.30	-0.05
75th	*3.65*	*2.35*	*1.32*	*0.14*	*0.28*	*0.59*	*0.44*	*0.02*
**80th**	4.79	2.73	1.50	0.63	0.37	0.68	0.50	0.10
**90th**	6.94	3.49	2.52	1.51	0.53	0.87	0.84	0.25
**95th**	8.14	3.98	3.23	2.60	0.63	1.00	1.08	0.43

Note: to produce cut-off values as a function of referent pain intensity and population percentiles, these intercept values are added to the corresponding model intercept values provided in Table 2, for example, for total PCS suing the 0-52 score range, model equation: PCS = 2.63*(pain intensity) + (0.2 + b_i). We operationally chose 75th percentile as an example cut-off to identify "high" catastrophizing.

When is Pain Catastrophizing not Catastrophizing?

Supplemental Materials

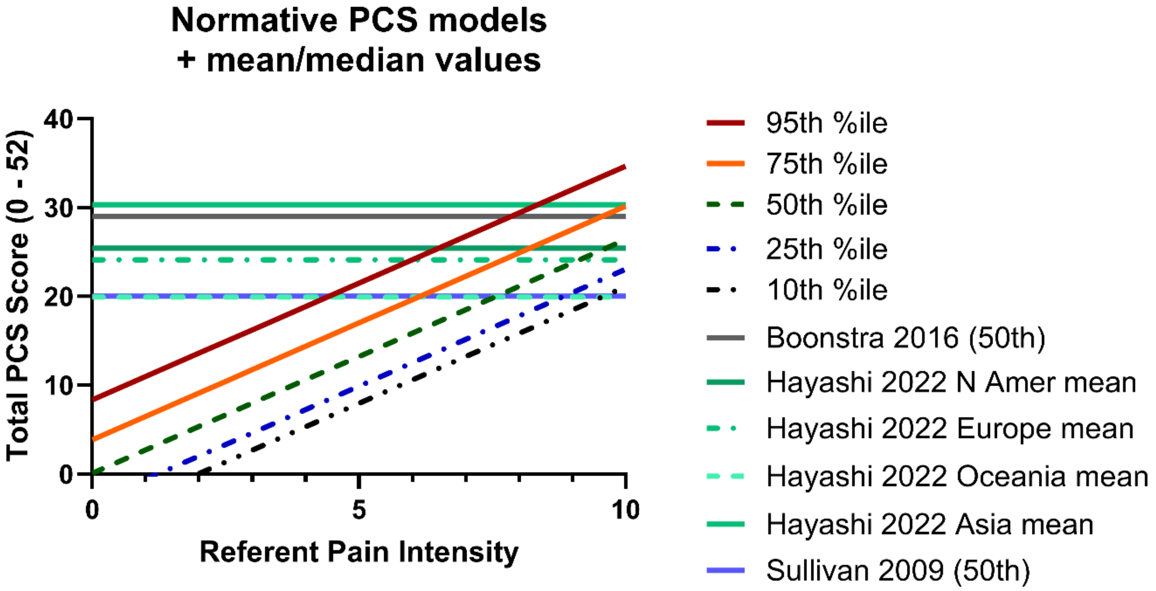

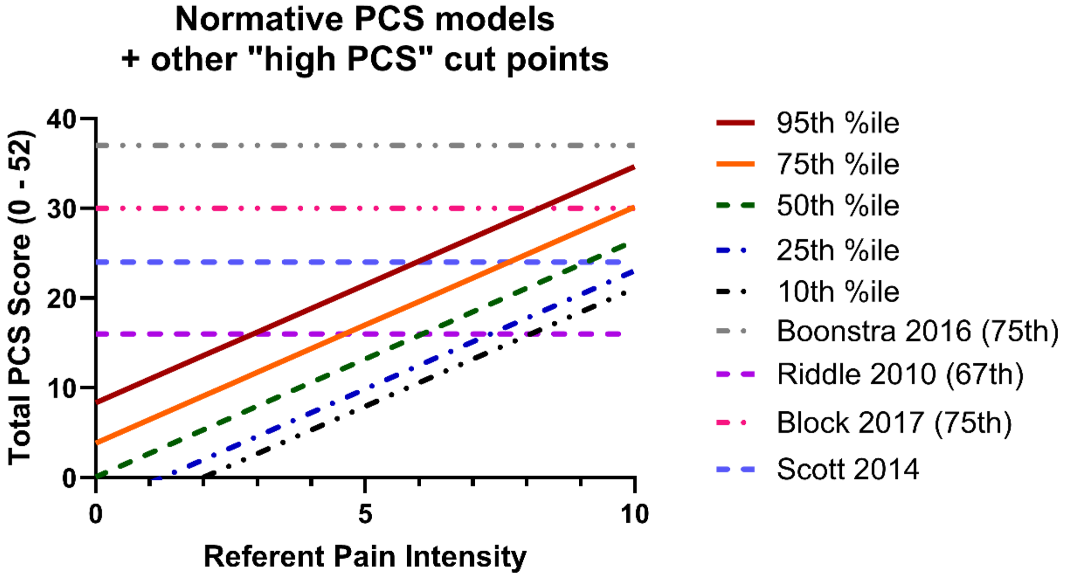

Figure S2. High PCS scores reported in the literature relative to the percentiles modeled in the current study between PCS and referent pain intensity. Each panel includes several examples of reported values for mean, median or higher percentiles in the literature. Note the wide range of values previously reported relative to the percentiles modeled for PCS scores relative to referent pain intensity in the current study.

When is Pain Catastrophizing not Catastrophizing?

Supplemental Materials

Table S2. Adjusted mixed linear model results (b, SE) with covariates with p-values < 0.05 explaining the variance in the study outcomes, PCS subscales, with and without standard subscale PCS scores as a covariate. Note that only the standardized, mean item subscale scores (range 0 – 4) are shown for easier comparison.

**Outcome** (range)	**Adjusted model with standard PCS subscale**			**Adjusted model without standard PCS subscale**		
	**Covariate Predictor**	**Beta (SE)**	**P-value**	**Covariate Predictor**	**Beta (SE)**	**P-value**
**PCS Ruminating** (0 – 4)	**Referent pain**	**0.25 (0.01)**	**< 2e-16**	**Referent pain**	**0.26 (0.01)**	**< 2e-16**
	**PCS-R (std)**	**0.48 (0.04)**	**< 2e-16**	**Max Prior Pain**	**-0.10 (0.03)**	**0.003**
	**Max Prior Pain**	**-0.07 (0.02)**	**0.003**	**SSAS**	**0.03 (0.009)**	**0.008**
	Current Pain (Y=1)	0.19 (0.08)	0.02			
	$mR^2 = 0.59$, $cR^2 = 0.68$			$mR^2 = 0.43$, $cR^2 = 0.68$		
**PCS Magnifying** (0 – 4)	**Referent pain**	**0.13 (0.01)**	**< 2e-16**	**Referent pain**	**0.14 (0.01)**	**< 2e-16**
	**PCS-M (std)**	**0.45 (0.05)**	**< 2e-15**	**NA**	**0.03 (0.009)**	**< 0.001**
	NA	0.02 (0.008)	0.03	SSAS	0.02 (0.009)	0.02
	Sex (m=0, f=1)	-0.17 (0.08)	0.04			
	$mR^2 = 0.43$, $cR^2 = 0.63$			$mR^2 = 0.30$, $cR^2 = 0.62$		
**PCS Helplessness** (0-4)	**Referent pain**	**0.18 (0.009)**	**< 2e-16**	**Referent pain**	**0.18 (0.009)**	**< 2e-16**
	**PCS-H (std)**	**0.37 (0.04)**	**< 2e-15**	**SSAS**	**0.02 (0.007)**	**< 0.001**
	Max Prior Pain	-0.05 (0.02)	0.02	NA	0.02 (0.007)	0.02
	SSAS	0.01 (0.006)	0.05			
	$mR^2 = 0.52$, $cR^2 = 0.61$			$mR^2 = 0.42$, $cR^2 = 0.61$		

Additional covariates included in each model but with p-values > 0.05: age, sex (m=0, f=1), negative affect (NA), maximum prior pain (0-10), mean pain rating schema value (PSI), positive affect, somatosensory amplification (SSAS), presence of current pain (yes/no). Note all covariates achieving p < 0.05 are shown, but only those achieving *a priori* level of p = 0.01 are in bold.

When is Pain Catastrophizing not Catastrophizing?

Supplemental Materials

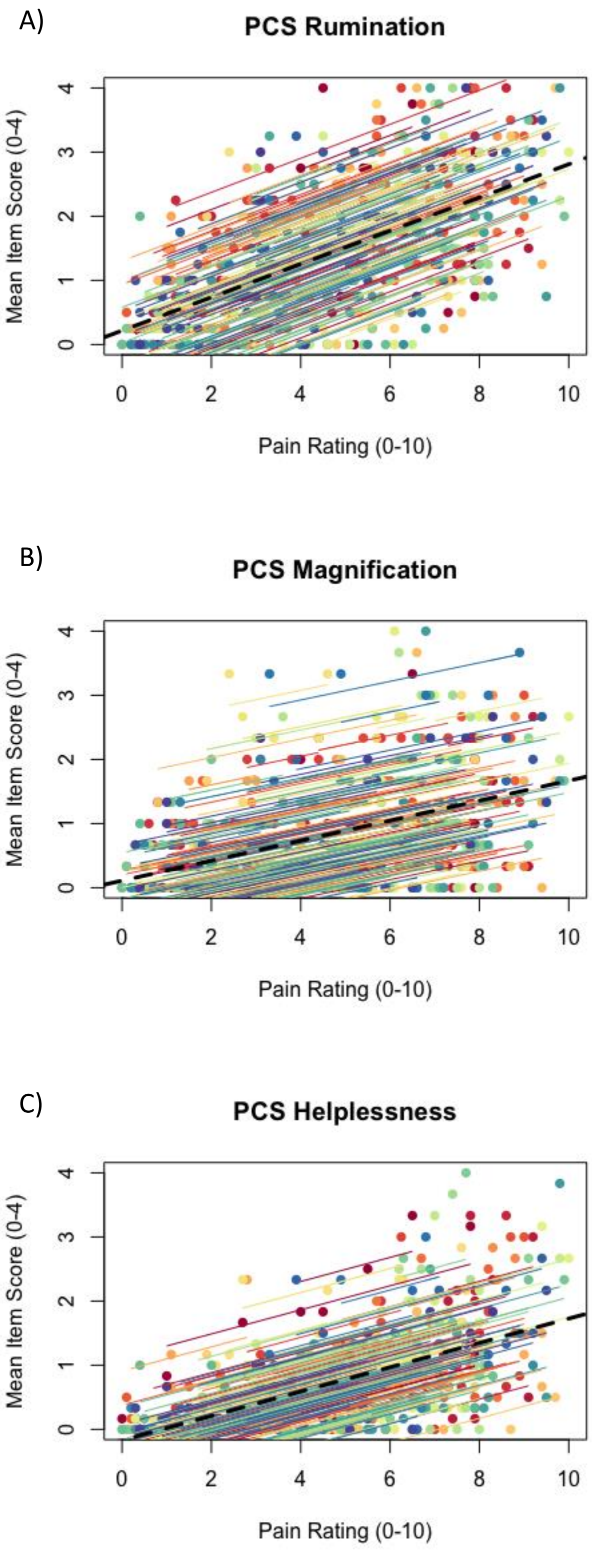

Figure S3. Scatterplot of individual pain catastrophizing (PCS) subscale standardized mean item scores relative to their referent pain scenario pain intensities, with the repeated measures modeled correlations for each individual (thin colored limes). The dark, dashed line represents the overall unadjusted relationship between each PCS subscale and referent pain intensity for A) Ruminating with R = 0.73 (95th percent confidence interval: 0.68 to 0.77, p < 0.00001), B) Magnifying with R = 0.55 (95th percent confidence interval: 0.47 to 0.61, p < 0.00001), and C) Helplessness with R = 0.67 (95th percent confidence interval: 0.62 to 0.73, p < 0.00001), using the repeated measures correlation (rmcorr) function in R.

When is Pain Catastrophizing not Catastrophizing?

Supplemental Materials

References

Boonstra A. M., Stewart R. E. , Köke A. J. A., Oosterwijk R. F. A., Swaan J. L., Schreurs K. M. G., Schiphorst Preuper H. R., (2016). Cut-off points for mild, moderate, and severe pain on the numeric rating scale for pain in patients with chronic musculoskeletal pain: variability and influence of sex and catastrophizing. *Frontiers in Psychology*, 7. doi=10.3389/fpsyg.2016.01466

Block, P. R., Thorn, B. E., Kapoor, S., & White, J. (2017). Pain catastrophizing, rather than vital signs, associated with pain intensity in patients presenting to the emergency department for pain. *Pain Management Nursing*, *18*(2), 102-109.

Hayashi, K., Ikemoto, T., Shiro, Y., Arai, Y.-C., Marcuzzi, A., Costa, D., et al. (2022). A systematic review of the variation in pain catastrophizing scale reference scores based on language version and country in patients with chronic primary (non-specific) pain. *Pain and Therapy,* 11(3), 753-769. doi: 10.1007/s40122-022-00390-0

Riddle DL, Wade JB, Jiranek WA, Kong X. (2010) Preoperative pain catastrophizing predicts pain outcome after knee arthroplasty. *Clin Orthop Relat Res*. 468(3), 798-806. doi: 10.1007/s11999-009-0963-y.

Scott W, Wideman TH, Sullivan MJ. (2014). Clinically meaningful scores on pain catastrophizing before and after multidisciplinary rehabilitation: a prospective study of individuals with subacute pain after whiplash injury. *Clin J Pain*. 30(3), 183-90. doi: 10.1097/AJP.0b013e31828eee6c.

Sullivan, M. J. L., Bishop, S. R., & Pivik, J. (1995). The Pain Catastrophizing Scale: Development and validation. Psychological Assessment, 7(4), 524–532. https://doi.org/10.1037/1040-3590.7.4.524
